# Effects of Complex Training on Sprint, Jump, and Change of Direction Ability of Soccer Players: A Systematic Review and Meta-Analysis

**DOI:** 10.3389/fpsyg.2020.627869

**Published:** 2021-01-22

**Authors:** Rohit K. Thapa, Danny Lum, Jason Moran, Rodrigo Ramirez-Campillo

**Affiliations:** ^1^Department of Sports Biomechanics, Lakshmibai National Institute of Physical Education, Gwalior, India; ^2^Sport Science and Sport Medicine, Singapore Sport Institute, Singapore, Singapore; ^3^Physical Education and Sport Science Academic Group, National Institute of Education, Nanyang Technological University, Singapore, Singapore; ^4^School of Sport Rehabilitation and Exercises Sciences, University of Essex, Colchester, United Kingdom; ^5^Human Performance Laboratory, Department of Physical Activity Sciences, Universidad de Los Lagos, Osorno, Chile; ^6^Centro de Investigación en Fisiología del Ejercicio, Facultad de Ciencias, Universidad Mayor, Santiago, Chile

**Keywords:** football, plyometric exercise, post-activation performance enhancement, physical education and training, resistance training, sports

## Abstract

The aim of this meta-analysis was to evaluate the effects of complex training (CT) on sprint, jump, and change of direction (COD) ability among soccer players. After an electronic search, 10 peer-reviewed articles were considered in the meta-analysis. The athletes included in this meta-analysis were amateur to professional level male soccer players (age range, 14–23 years). These studies incorporated CT in soccer players who were compared to a control group. Significant moderate to large improvements were observed in the CT group [sprint: standard mean difference (SMD) = 0.92–1.91; jump: SMD = 0.96–1.58; COD: SMD = 0.97–1.49] when compared to control groups. Subgroup analysis were also conducted based on age, duration, and competitive level. The beneficial effects of CT were greater in players <18 vs. ≥18 years (linear sprinting; SMD = 2.01 vs. −0.13), after ≥8 vs. <8 weeks (jumping and COD; SMD = 1.55–2.01 vs. 0.31–0.64, respectively) and among professional vs. amateur players (linear sprinting and with COD; SMD = 1.53–1.58 vs. 0.08–0.63, respectively). In conclusion, regular soccer training programs may be supplemented with CT to improve sprint, jump, and COD performance. A longer duration of CT (≥8 weeks) seems to be optimal in improving the physical abilities of soccer players. Professional players and <18 years players may benefit more from CT program.

## Introduction

Soccer is a team sport which requires players to execute several short-duration maximal- and near-maximal physical efforts such as sprinting, jumping, and change(ing) of direction (COD) in order to overcome opponents during play (Stølen et al., 2005; Barnes et al., 2014). These physical fitness abilities are crucial in determining a player's performance potential during soccer games and tournaments (Castagna et al., 2003; Arnason et al., 2004; Faude et al., 2012), and have been linked with strength and power of the lower extremities (Arnason et al., 2004; Wisløff et al., 2004). To increase strength and power, resistance training (RT) and plyometric (PT)/power training programs are widely used in soccer (De Hoyo et al., 2016; Bauer et al., 2019; Ramirez-Campillo et al., 2020b). The aforementioned training methods induce neuromuscular adaptations, such as enhanced stretch-shortening cycle function, motor unit recruitment, firing frequency, intra- and inter-muscular coordination, and morphological changes (e.g., fiber type or pennation angle), enhancing overall performance (Markovic and Mikulic, 2010; Cormie et al., 2011). Although both RT (Marios et al., 2006; Sander et al., 2013; Silva et al., 2015) and PT (Van De Hoef et al., 2019; Ramirez-Campillo et al., 2020a,b) may improve strength and power, respectively, conducting independent training sessions for each method within a congested weekly micro-cycle may be troublesome for players and coaches.

Alternatively, a pragmatic approach combines both RT and PT methods within a single training work set, allowing more time to be devoted to specific soccer training activities. One such combination is termed as complex training (CT) (Fleck and Kontor, 1986), which commonly involves the performance of an exercise set with a high-load RT exercise, followed immediately by the execution of a low-load plyometric-power exercise. This format of training usually involves the combination of two biomechanically similar exercises with the high-load resistance exercise performed first [e.g., squat at 90% of one repetition maximum (1RM)], followed by the low-load power exercise (e.g., squat jumps) (Fleck and Kontor, 1986; Docherty et al., 2004). Such exercise sequencing stimulates the post-activation potentiation of performance, a phenomenon (Docherty et al., 2004; Hodgson et al., 2005; Carter and Greenwood, 2014; Prieske et al., 2020), that stimulates motor unit recruitment thus increasing the force producing potential of the utilized musculature within a given movement (Healy and Comyns, 2017).

In the last four decades (Fleck and Kontor, 1986), a considerable amount of published studies have analyzed the effects of CT on athletes' physical fitness and some meta-analyses have attempted to congregate the extant literature (Freitas et al., 2017; Bauer et al., 2019; Cormier et al., 2020; Pagaduan and Pojskic, 2020). Some meta-analyses have also been conducted specifically on team sports athletes (Freitas et al., 2017; Cormier et al., 2020). However, these studies included athletes with different sporting backgrounds (i.e., soccer, futsal, basketball, volleyball, rugby, football, water polo, track and field, and handball), potentially biasing the results for a particular sport, such as soccer. Indeed, the effects of PT and RT may vary according to an athlete's athletic history (Izquierdo et al., 2002; Fleck and Kraemer, 2004; De Villarreal et al., 2012; Asadi et al., 2016; Taylor et al., 2018; Vlachopoulos et al., 2018). Moreover, a 5-fold difference may be observed in the magnitude of the effects of CT on the physical fitness performance (i.e., sprinting) of athletes with different team sport background (Freitas et al., 2017).

Therefore, in order to clarify the specific effects of CT (and its moderators; e.g., programme duration) on soccer player's physical fitness performance, the aim of this meta-analysis was to evaluate the effects of CT interventions on sprint, jump, and COD ability among soccer players. With reference to previous studies (Freitas et al., 2017; Kobal et al., 2017; Bauer et al., 2019; Cormier et al., 2020), we hypothesized that CT would be effective in improving soccer players' physical fitness, with greater effects observed as compared to a control condition. Accordingly, our research question was: what are the effects of CT interventions (and its moderators) on sprint, jump, and COD ability among soccer players when compared to a control group?

## Methods

The lead investigator (RKT) and a research assistant conducted electronic searches. The PubMed and Google scholar databases were used, conforming to the guidelines set by the Preferred Reported Items for Systematic Reviews and Meta-Analysis (PRISMA) (Moher et al., 2015). This study was registered with the International Platform of Registered Systematic Review and Meta-Analysis Protocols (INPLASY202090079). Articles published up to August 30th, 2020 were considered. Keywords were selected through experts' opinion and a systematic literature review. Using Boolean logic, the following combination of keywords was used in the search databases: “complex training” or “contrast training” or “combination of strength training and plyometrics” and “soccer” or “football.” An example for search strategy used in PubMed was: [(((complex training) OR contrast training) OR (combination of strength training and plyometrics)) AND soccer] OR football. Relevant articles' reference lists were examined to identify further articles for inclusion in the meta-analysis.

### Inclusion Criteria and Exclusion Criteria

A PICOS (participants, intervention, comparators, outcomes, and study design) approach was used to rate studies' eligibility (Liberati et al., 2009). The respective inclusion/exclusion criteria adopted in our meta-analysis are reported in Table 1.

**Table 1:** Selection criteria used in the meta-analysis.

**Category**	**Inclusion criteria**	**Exclusion criteria**
Population	Apparently healthy soccer players, with no restrictions on their playing level, sex, or age	Soccer players with health problems (e.g., injuries, recent surgery)
Intervention	A complex training programme, defined as a combination of heavy load strength exercise followed by a low load plyometric/power exercise, set by set	Exercise interventions not involving complex training or exercise interventions involving contrast training, where strength training exercises were conducted first and plyometric/power exercises at the end of the session (or during a different session)
Comparator	Active control group	Absence of active control group
Outcome	At least one measure of physical fitness (linear sprinting, jumping, and change of direction speed) before and after the training intervention	Lack of baseline and/or follow-up data
Study design	Controlled trials	Non-controlled trials

### Methodological Quality of Studies

The Tool for the Assessment of Study Quality and Reporting in Exercise (TESTEX) was used to assess the quality of studies included in this meta-analysis (Smart et al., 2015). This tool comprises of 12 methodological quality assessment criteria (a full description of each criteria is provided in the results section for the included studies). The maximum score a study can obtain is 15 points, with higher ratings reflecting better study quality and reporting (Smart et al., 2015).

### Data Extraction

We extracted from each eligible study, data relating to linear sprints over various distances (5–40-m linear sprint tests), vertical jumping and sprints which incorporated a COD (505, S4 × 5, *T*-test, BAT, S180 tests and dribbling with or without ball) (Castagna et al., 2003; Arnason et al., 2004; Faude et al., 2012). Means, standard deviations (SD), and sample sizes (*n*) (Table 2) were extracted by one author (RKT) from the included papers and were corroborated by a second author (DL). Any discrepancy between the authors was resolved through discussion with a third author (RRC). Where data were displayed in a figure or no numerical data were provided by authors after being contacted, validated (*r* = 0.99, *p* < 0.001) software (WebPlotDigitizer; https://apps.automeris.io/wpd/) was used to derive numerical data from figures (Drevon et al., 2017). In addition to the study data, sample characteristics, including age, playing level, training frequency, total duration of the intervention, and the type of training protocol used in the study were extracted and recorded.

**Table 2:** The mean ± standard deviation of fitness variables reported for the complex training and control conditions in the included studies.

**References**	**Fitness attribute**	**Complex training**			**Control**		
		**Pre**	**Post**	***n***	**Pre**	**Post**	***n***
Ali et al. (2019)	20m (s) CMJ (cm) *T*-test (s)	3.48 ± 0.35 46.98 ± 4.84 11.72 ± 1.11	3.23 ± 0.26 50.75 ± 4.91 11.15 ± 0.94	12 12 12	3.40 ± 0.26 43.97 ± 6.92 11.60 ± 1.01	3.54 ± 0.27 44.35 ± 6.92 11.73 ± 1.16	12 12 12
Maio Alves et al. (2010) (1 session/week)	5m (s) 15m (s) SJ (cm) CMJ (cm) 505 (s)	1.09 ± 0.07 2.56 ± 0.10 41.02 ± 6.11 42.84 ± 4.55 2.34 ± 0.11	0.99 ± 0.03 2.38 ± 0.09 46.19 ± 7.70 42.92 ± 5.56 2.31 ± 0.09	9 9 9 9 9	1.13 ± 0.08 2.59 ± 0.07 41 ± 3.05 42.63 ± 3.35 2.37 ± 0.09	1.11 ± 0.03 2.56 ± 0.02 40.7 ± 3.91 41.53 ± 2.71 2.39 ± 0.15	6 6 6 6 6
Maio Alves et al. (2010) (2 sessions/week)	5m (s) 15m (s) SJ (cm) CMJ (cm) 505 (s)	1.13 ± 0.04 2.57 ± 0.10 39.68 ± 4.34 41.78 ± 5.28 2.32 ± 0.08	1.06 ± 0.03 2.49 ± 0.07 43.5 ± 4.50 42.79 ± 4.45 2.32 ± 0.03	8 8 8 8 8	1.13 ± 0.08 2.59 ± 0.07 41 ± 3.05 42.63 ± 3.35 2.37 ± 0.09	1.11 ± 0.03 2.56 ± 0.02 40.7 ± 3.91 41.53 ± 2.71 2.39 ± 0.15	6 6 6 6 6
Brito et al. (2014)	5m (s) 20m (s)	1.13 ± 0.02 3.25 ± 0.09	1.02 ± 0.02 3.05 ± 0.07	12 12	1.14 ± 0.02 3.21 ± 0.13	1.11 ± 0.02 3.17 ± 0.12	26 26
Cavaco et al. (2014) (1 session/week)	15m (s) Agility with ball (s)	2.72 ± 0.25 10.64 ± 1.82	2.59 ± 0.18 9.80 ± 1.43	5 5	2.68 ± 0.19 9.88 ± 0.48	2.66 ± 0.18 9.90 ± 0.46	6 6
Cavaco et al. (2014) (2 sessions/week)	15m (s) Agility with ball (s)	2.6 ± 0.14 9.64 ± 1.23	2.46 ± 0.15 8.54 ± 0.37	5 5	2.68 ± 0.19 9.88 ± 0.48	2.66 ± 0.18 9.90 ± 0.46	6 6
Chatzinikolaou et al. (2018)	10m (s) 30m (s) CMJ (cm) CMJ arm swing (cm) SJ (cm)	1.90 ± 0.08 4.8 ± 0.43 35.84 ± 4 42.33 ± 4.74 32.95 ± 4.19	1.83 ± 0.07 4.32 ± 0.21 38.2 ± 3.84 45.4 ± 4.37 34.95 ± 3.81	12 12 12 12 12	1.93 ± 0.12 4.69 ± 0.43 35.03 ± 3.55 41.49 ± 3.07 32.29 ± 3.9	2.02 ± 0.12 4.79 ± 0.44 32.08 ± 2.67 38.33 ± 1.76 30.1 ± 3.61	10 10 10 10 10
Faude et al. (2013)	10m (s) 30m (s) CMJ (cm) COD (s)	1.77 ± 0.07 4.26 ± 0.15 40.2 ± 4.8 9.8 ± 0.7	1.77 ± 0.05 4.27 ± 0.09 41.4 ± 3.5 9.4 ± 0.4	8 8 8 8	1.73 ± 0.08 4.12 ± 0.10 43 ± 2.1 9.5 ± 0.04	1.77 ± 0.05 4.19 ± 0.09 39.8 ± 2.4 9.3 ± 0.3	8 8 8 8
García-Pinillos et al. (2014)	5m (s) 10m (s) 20m (s) 30m (s) CMJ (cm) BAT (s)	1.67 ± 0.23 2.47 ± 0.25 3.83 ± 0.29 5.11 ± 0.35 42 ± 6 12.29 ± 0.54	1.42 ± 0.18 2.14 ± 0.30 3.51 ± 0.24 4.79 ± 0.30 45 ± 4 11.66 ± 0.43	17 17 17 17 17 17	1.66 ± 0.17 2.42 ± 0.15 3.74 ± 0.17 4.96 ± 0.18 45 ± 3 11.93 ± 0.58	1.46 ± 0.18 2.24 ± 0.17 3.53 ± 0.21 4.77 ± 0.24 46 ± 3 11.89 ± 0.46	13 13 13 13 13 13
Hammami et al. (2017a)	5m (s) 10m (s) 20m (s) 30m (s) 40m (s) SJ (cm) CMJ (cm) S180 (s) S4x5 (s)	1.14 ± 0.03 1.87 ± 0.23 3.32 ± 0.17 4.57 ± 0.14 5.96 ± 0.21 36.8 ± 2.6 38.4 ± 1.9 8.37 ± 0.21 6.28 ± 0.25	1.01 ± 0.02 1.73 ± 0.13 3.04 ± 0.04 4.23 ± 0.06 5.42 ± 0.15 45.4 ± 3.5 48.1 ± 4.5 7.93 ± 0.26 5.83 ± 0.04	16 16 16 16 16 16 16 16 16	1.15 ± 0.09 1.92 ± 0.13 3.30 ± 0.22 4.59 ± 0.33 5.94 ± 0.41 35.4 ± 5.1 3.7 ± 6.8 8.41 ± 0.33 6.29 ± 0.21	1.12 ± 0.06 1.90 ± 0.14 3.30 ± 0.23 4.56 ± 0.33 5.85 ± 0.42 34.3 ± 5.2 36.1 ± 5.9 8.40 ± 0.35 6.26 ± 0.16	12 12 12 12 12 12 12 12 12
Hammami et al. (2017b)	5m (s) 10m (s) 20m (s) 30m (s) 40m (s) S180 (s) S4x5 (s)	1.14 ± 0.04 1.91 ± 0.4 3.30 ± 0.17 4.56 ± 0.14 5.93 ± 0.21 8.37 ± 0.21 6.24 ± 0.26	1.01 ± 0.02 1.76 ± 0.04 3.04 ± 0.05 4.23 ± 0.08 5.43 ± 0.16 7.94 ± 0.25 5.84 ± 0.05	17 17 17 17 17 17 17	1.06 ± 0.12 1.87 ± 0.16 3.21 ± 0.25 4.51 ± 0.37 5.90 ± 0.40 8.39 ± 0.35 6.18 ± 0.29	1.11 ± 0.11 1.90 ± 0.17 3.27 ± 0.25 4.56 ± 0.37 5.81 ± 0.43 8.40 ± 0.37 6.13 ± 0.22	12 12 12 12 12 12 12
Hammami et al. (2019)	5m (s) 40m (s) SJ (cm) CMJ (cm) S4x5 (s)	1.13 ± 0.04 5.93 ± 0.22 36.8 ± 2.8 38.2 ± 2.1 6.25 ± 0.26	1.01 ± 0.17 5.43 ± 0.17 45.4 ± 3.7 47 ± 5.9 5.83 ± 0.47	14 14 14 14 14	1.03 ± 0.06 5.85 ± 0.32 36.2 ± 3.8 37.9 ± 5.4 6.15 ± 0.25	1.08 ± 0.04 5.75 ± 0.33 34.8 ± 4.5 37.2 ± 4.5 6.61 ± 0.26	12 12 12 12 12

*5m, 5-m linear sprint time; 10m, 10-m linear sprint time; 20m, 20-m linear sprint time; 30m, 30-m linear sprint time; 40m, 40-m linear sprint time; BAT, Balsom agility test; COD, change of direction; CMJ, countermovement jump; CMJ arm swing, countermovement jump with arm swing; SJ, squat jump; S180, Sprinting 9-3-6-3-9 m with 180° Turns; S4 × 5, 4 × 5m sprints with COD*.

### Statistical Analysis

The meta-analysis was conducted using the Review Manager Software (RevMan 5.3). Statistical significance was set at *p* ≤ 0.05. The inverse-variance random effects model for meta-analyses was used because it allocates a proportionate weight to trials based on the size of their individual standard errors (Deeks et al., 2008) and facilitates analysis whilst accounting for heterogeneity across studies (Kontopantelis et al., 2013). Effect sizes are represented by the standardized mean difference and are presented alongside 95% confidence intervals (CI). Standardized mean differences were calculated using the following equation (Cohen, 1988):

$$\text{SMD}=\frac{\text{M1}-\text{M2}}{\text{SD pooled}}$$

Where M1 - M2 is the difference in the mean outcome between groups, and SD pooled is the pooled standard deviation of the outcome among subjects, which was calculated using the following equation:

$$\begin{array}{l} SD\;pooled=\sqrt{\frac{SD_{1}^{2}+SD_{2}^{2}}{2}} \end{array}$$

Where $SD_{1}^{2}$ is the squared standard deviation of group 1 and $SD_{2}^{2}$ is the squared standard deviation of group 2.

The SMD were interpreted with threshold values as follow: <0.2, trivial; 0.2–0.6, small; >0.6–1.2, moderate; >1.2–2.0, large; >2.0–4.0, very large; >4.0, extremely large (Hopkins et al., 2009).

In studies with more than one intervention group, the control group was proportionately divided to facilitate comparison across all participants (Higgins et al., 2008).

Subgroup analyses were performed on the variables that could have influenced the outcome after CT interventions, with median values of continues variables used as cut-off values for grouping [age (<18 vs. ≥18 years), and CT programme duration (<8 vs. ≥8 weeks)]. The subgrouping of studies according to the athlete's competition level [professional vs. amateur (youth, university); as reported in the studies] was also considered. The analyses were conducted when there were at least one study in the subgroups.

The *I*^2^ statistics was used to assess the heterogeneity among the studies, with values of <25, 25–75, and >75% interpreted as low, moderate and high heterogeneity, respectively (Higgins et al., 2003; Higgins, 2008). Heterogeneity was assessed in line with a low chi-squares' *p*-value (Higgins et al., 2003; Higgins, 2008) and a high Tau^2^ value (Borenstein et al., 2009).

## Results

**Figure 1:**
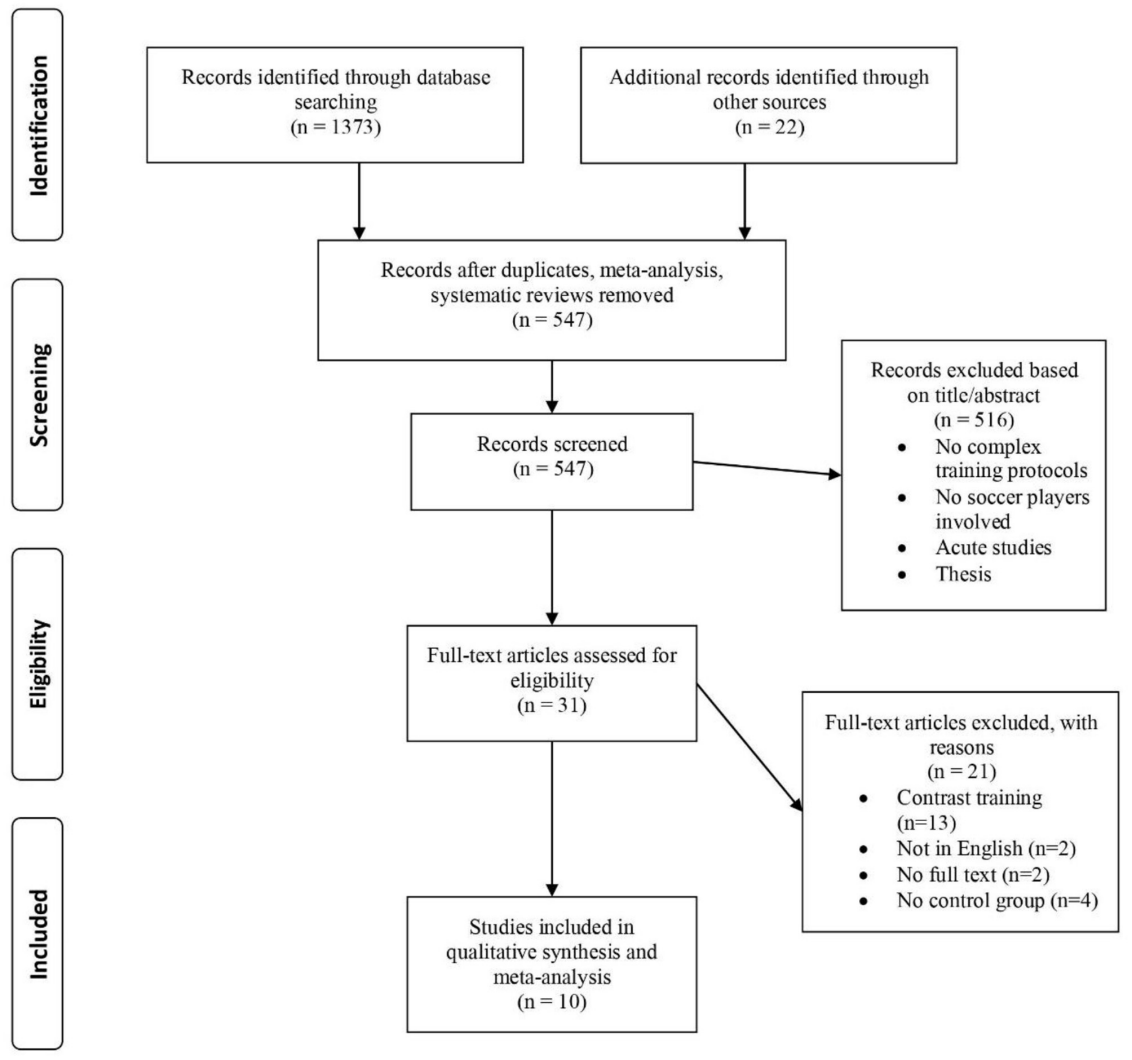
Flow diagram of study selection process.

The initial search resulted in the retrieval of 1,373 articles and 22 additional articles were extracted through other sources. After removing duplicates, meta-analysis and systematic reviews 547 articles remained. After screening titles and abstracts for relevance, 516 articles were excluded with 31 full texts being retained. Further screening of articles based on our inclusion and exclusion criteria resulted in the inclusion of 10 studies in the meta-analysis (Maio Alves et al., 2010; Faude et al., 2013; Brito et al., 2014; Cavaco et al., 2014; García-Pinillos et al., 2014; Hammami et al., 2017a,b, 2019; Chatzinikolaou et al., 2018; Ali et al., 2019) (Figure 1).

### General Characteristics of Studies

The study characteristics are presented in Table 3. A total of 313 subjects (all males) were included in this meta-analysis with 188 professional players and 125 amateur players. Sprinting time was measured through 5–40-m linear sprint tests. Jumping was measured using jump height in the squat jump and countermovement jump. The sprint time with COD was measured among the included studies using the 505, S4 × 5, *T*-test, BAT, and S180 tests, with or without dribbling a soccer ball. Considering the different number of COD (i.e., turns) involved among the tests, in *a posteriori* decision the tests were further divided based on those including ≤ 3 turns and >3 turns. Accordingly, the 505 and S4 × 5 tests involved ≤ 3 turns, while the *T*-test, BAT, and S180 tests involved >3 turns. Two studies assessed COD with ball, using ≤ 3 turns (Faude et al., 2013) and >3 turns (Cavaco et al., 2014).

**Table 3:** Study characteristics.

**References**	**Groups**	**Subjects (*n*)**	**Age (Mean ± SD)**	**Training intervention**				**Variables**
				**Level**	**Frequency/wk**.	**Duration (weeks)**	**Training protocol**	
Maio Alves et al. (2010)	CT1 CT2 CG	9 8 6	17.4 ± 0.6	P	1 2	6	**Pre-season** 1st station: 6 rep of 90 squat exercise at 85% 1RM + 1 set of 5 m high skipping in straight line and then 5 m sprint. 2nd station: 6 rep of calf extension exercise at 90% 1RM + 8 vertical jumps + 3 high-ball headers. 3rd station: 6 rep of leg extension exercise at 80% 1RM + 6 jumps from the seated position + 3 drops jumps (60 cm) executing a soccer heading. The load was increased 5% (from 1RM) every 2 weeks.	5m, 15m, SJ, CMJ, COD
Ali et al. (2019)	CT CST CG	12 12 12	22 ± 2.4 20.8 ± 2.1 21.5 ± 1.8	C	3	6	**Off-season** Squats (80% 1 RM) 3 × 12 + Depth jumps 3 × 12 Barbell lunge (80% 1 RM) 3 × 12 + Split squat jumps 3 × 12 Lateral lunge (80% 1 RM) 3 × 12 + Lateral hops 3 × 12	20m, CMJ, COD (*T*-test)
Brito et al. (2014)	CG ST PT CT	21 12 12 12	20.3 ± 1.6	C	2	9	**In-season** Station 1: 6 reps of 85% of 1RM SQ at 90°+ 1 set high skipping, cyclically, with thighs Parallel to the ground (5m) + 1 5m sprint Station 2: 6 reps at 90% of 1RM calf extension + 8 consecutive vertical jumps withMinimum GCT + 3 ball headers (jumping as high as possible) Station 3: 6 reps at 80% of 1RM of leg extension + 6 vertical jumps from seated position+ 3 DJ (60 cm) with soccer heading post vertical jumps	5m, 20m, SJ, CMJ, COD (*T*-test)
Cavaco et al. (2014)	CT1 CT2 CG	5 5 6	13.8 ± 0.4 14.2 ± 0.4 14.2 ± 0.8	Y	1 2	6	First station: 3 sets, with 6 squat repetitions at 85% of 1-RM, 15 m plus top speed and cross, separated by 3 min of rest. Second station: 3 sets, 6 repetitions of the squat at 85% of 1-RM, plus agility with the ball and shot at the goal, separated by 3 min of rest. The load in the squat exercise was increased by 5% from initial 1RM each 2 weeks.	15m, 15m agility with ball
Chatzinikolaou et al. (2018)	CG CT	10 12	14.1 ± 0.6 14.3 ± 0.7	P	2	5	**Off-season** 2 days ST with 13 exercises 2 days CXT Day 1: Barbell cleans+10–20m sprints/agility drills; Kettlebell snatch+10–20m sprints/agility drills; Box jumps+10m agility drills Day 2: Power bag jerk+10–20m sprints; Kettlebell snatch_10–20m sprints; Resistive sprints + sprints (8m + 15–20m)	10m, 30m, SJ, CMJ, COD (Arrowhead)
Faude et al. (2013)	CG CT	8 8	22.5 ± 2.5	A	2	7	**In-season** Day 1: Unilateral load half SQ (90% of estimated 1 RM) + 5 single leg hurdle jumps (right and left leg) Day 2: (50–60% 1RM)	10m, 30m, CMJ, COD
							5 loaded half SQ + 5 DJ + 5m sprint; 5 loaded calf raises + 5 high straight jumps + 1 headers; 8 loaded lateral half SQ (4 left, 4 right side) + 8 lateral jumps + 10 zig-zag sprints; 8 loaded step ups (4 left, 4 right side) + 4 bounding jumps + 3 headers	
García-Pinillos et al. (2014)	CG CT	13 17	15.9 ± 1.4	P	2	12	**In-season** Weeks 1–2: 90° isometric half SQ (40 s) + jump from seated position (6 reps) Weeks 3–4: 90° isometric half SQ (60 s) + alternate single leg jump (6 reps) Weeks 5–6: 90° isometric half SQ (80 s) + jump from seated position/ alternate single leg jump (alternatively) Weeks 7–8: 90° isometric half SQ (40 s) + alternate single leg jump (6 reps) + 90° isometric half SQ (40 s) + jump from seated position (6 reps) Week 9–10: 90° isometric half SQ (40 s) + alternate single leg jump (6 reps) + 90° isometric half SQ (40 s) + jump from seated position (6 reps) Week 11–12: same as 9–10	5m, 10m, 20m, 30m, CMJ, COD (BAT)
Hammami et al. (2017a)	CG ST CT	12 16 16	16 ± 0.5	P	2	8	**In-season** First 4 weeks: Back Half SQ+3 consecutive CMJ Second 4 weeks: Back Half SQ+1 CMJ+15m sprint	5m, 10m, 20m, 30m, 40m, SJ, CMJ, S180, S4 × 5m
Hammami et al. (2017b)	CT CG	17 12	16.0 ± 0.5 16.8 ± 0.2	P	2	8	**In-season** Weeks 1–4: Half SQ + 3 consecutive CMJ Weeks 5–8: Half SQ + 1 CMJ + 15m sprint	5m, 10m, 20m, 30m, 40m, S4x5, S180
Hammami et al. (2019)	CT PT CG	14 14 12	15.8 ± 0.4	P	2	8	**In-season** Weeks 1–4: Half SQ + 3 consecutive CMJ Weeks 5–8: Half SQ + 1 CMJ + 15m sprint	5m, 40m, SJ, CMJ, COD (4 × 5m)

*1RM, one repetition maximum; 5m, 5-m linear sprint time; 10m, 10-m linear sprint time; 15m, 15-m linear sprint time; 20m, 20-m linear sprint time; 30m, 30-m linear sprint time; 40m, 40-m linear sprint time; A, amateur soccer team; BAT, Balsom agility test; C, college level; CG, control Group; CMJ, countermovement jump; COD, change of direction; CT, complex training group; CT1, complex training with one session per week; CT2, complex training with two sessions per week; CST, contrast training group; DJ, drop jump; P, professional soccer team. Some youth teams were categorized as professionals, considering the training load and/or playing level described in the respective studies (e.g., “Elite”); PT, plyometric training group; RDL, Romanian deadlift; rep, repetitions; S, semi-professional; S180, Sprinting 9-3-6-3-9 m with 180° Turns; S4 × 5, 4 × 5m sprints with COD; SJ, squat jump; SPT, sprint training group; SQ, squat; Y, youth*.

### Methodological Quality of Included Studies

Table 4 shows the methodological quality of the eligible studies in this meta-analysis. The score of all the studies according to TESTEX criteria ranged from 10 to 13 points. All studies selected for the meta-analysis obtained a score ≥10 points. Therefore, no study was excluded based on its methodological quality.

**Table 4:** Methodological quality score of the studies included in the meta-analysis.

**References**	**Items**												**Total points (from a maximum of 15)**
	**1**	**2**	**3**	**4**	**5**	**6**	**7**	**8**	**9**	**10**	**11**	**12**	
Ali et al. (2019)	1	–	–	1	–	1	1	2	1	1	1	1	10
Maio Alves et al. (2010)	1	–	1	1	–	1	1	2	1	1	1	1	11
Brito et al. (2014)	1	1	1	1	–	1	1	2	1	1	1	1	12
Cavaco et al. (2014)	1	1	1	1	–	1	1	2	1	1	1	1	12
Chatzinikolaou et al. (2018)	1	1	1	1	–	2	1	2	1	1	1	1	13
Faude et al. (2013)	1	1	1	–	–	2	1	2	1	1	1	1	12
García-Pinillos et al. (2014)	1	1	1	1	–	1	1	2	1	1	1	1	12
Hammami et al. (2017a)	1	1	1	–	–	1	1	2	1	1	1	1	11
Hammami et al. (2017b)	1	1	1	1	–	1	1	2	1	1	1	1	11
Hammami et al. (2019)	1	1	1	1	–	1	1	2	1	1	1	1	12

*Note for items 1 through 12: (1) eligibility criteria specified (1 point); (2) randomization defined (1 point); (3) allocation concealment (1 point); (4) groups similar at baseline (1 point); (5) assessor blinding in study reporting (1 point); (6) outcome measures assessed in 85% of patients (3 points); (7) intention-to-treat analysis (1 point); (8) between-group statistical comparisons reported (2 points); (9) point measures and measures of variability for all reported outcome measures (1 point); (10) activity monitoring in the controlled group (1 point); (11) relative exercise intensity remained constant (1 point); (12) exercise volume and energy expenditure (1 point)*.

### Meta-Analysis Results for Linear Sprint Performance

A moderate to very large significant improvement was noted after CT (within group, pre-post analysis) for 5-m (SMD = 2.44, 95% CI = 1.34–3.54, *p* < 0.001), 10-m (SMD = 0.67, 95% CI = 0.34–0.99, *p* < 0.001), 15-m (SMD = 1.05, 95% CI = 0.46–1.64, *p* < 0.001), 20-m (SMD = 1.43, 95% CI = 0.74–2.12, *p* < 0.001), 30-m (SMD = 1.31, 95% CI = 0.27–2.35, *p* = 0.01), and 40-m linear sprint performance (SMD = 2.65, 95% CI = 2.08–3.23, *p* < 0.001). The relative weight of each study in the analyses varied between 10.8 and 37.7%, with low to high heterogeneity (*I*^2^ = 0–88.0%).

**Figure 2:**
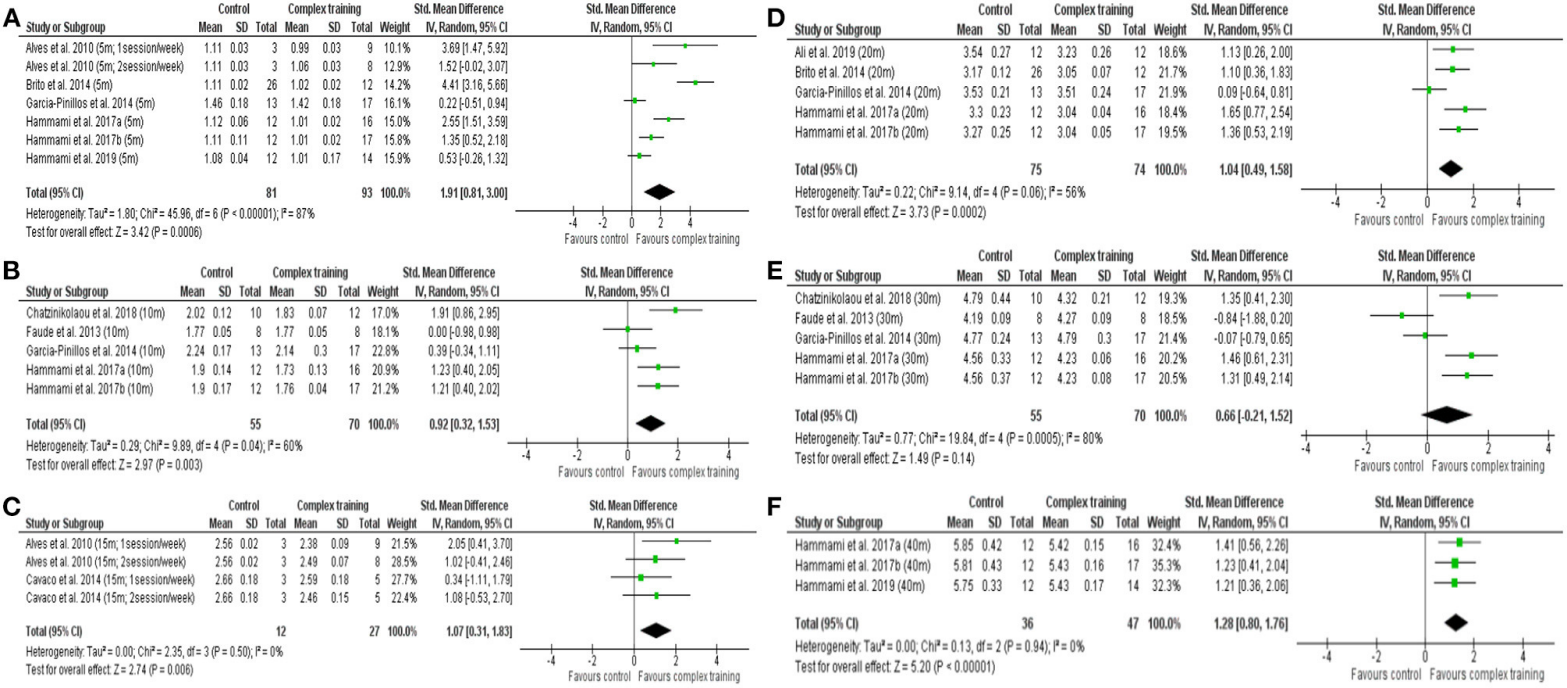
Forest plot of standardized mean difference in post-intervention outcomes between complex training group and control group, including **(A)** 5-m sprint time, **(B)** 10-m sprint time, **(C)** 15-m sprint time, **(D)** 20-m sprint time, **(E)** 30-m sprint time, **(F)** 40-m sprint time. Note: the relative weight of each study in the analysis is indicated by the size of the green squares.

A moderate to large significant difference was noted between CT and control (between group, post analysis) for 5-m (SMD = 1.91, 95% CI = 0.81–3.00, *p* < 0.001, Figure 2A), 10-m (SMD = 0.92, 95% CI = 0.32–1.53, *p* = 0.003, Figure 2B), 15-m (SMD = 1.07, 95% CI = 0.31–1.83, *p* = 0.006, Figure 2C), 20-m (SMD = 1.04, 95% CI = 0.49–1.58, *p* < 0.001, Figure 2D), and 40-m linear sprint performance (SMD = 1.28, 95% CI = 0.80–1.76, *p* < 0.001, Figure 2F). The relative weight of each study in the analyses varied between 10.1 and 35.3%, with low to high heterogeneity (*I*^2^ = 0–87%). Only 30-m linear sprint showed no difference between CT and control (Figure 2E).

### Meta-Analysis Results for Vertical Jump Performance

A moderate to large significant improvement was noted after CT (within group, pre-post analysis) for countermovement jump (SMD = 0.89, 95% CI = 1.43–0.35, *p* < 0.001) and squat jump height performance (SMD = 1.33, 95% CI = 2.12–0.54, *p* = 0.001). The relative weight of each study in the analyses varied between 10.4 and 17.9%, with low to high heterogeneity (*I*^2^ = 64.0–76.0%).

**Figure 3:**
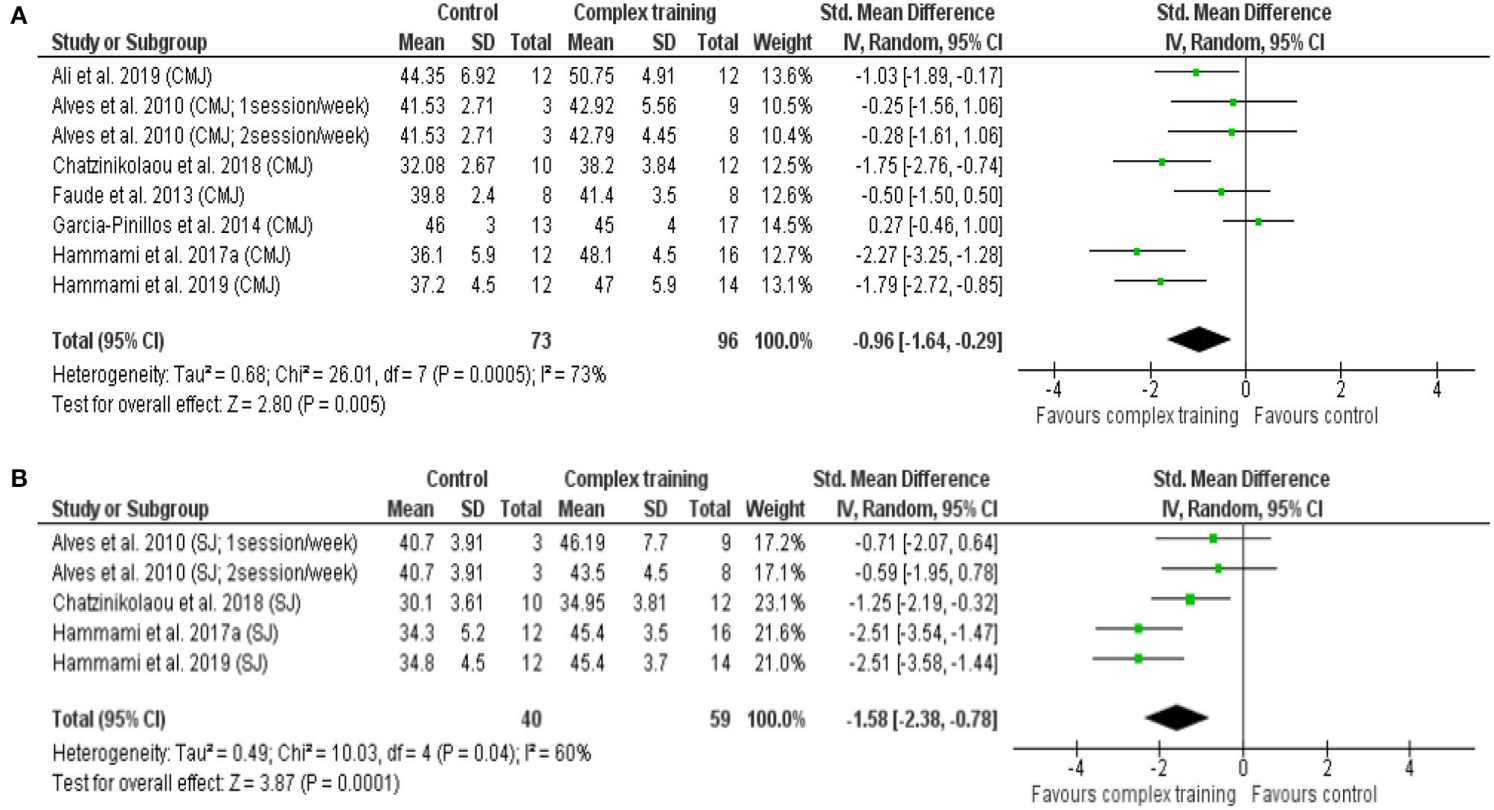
Forest plot of standardized mean difference in post-intervention outcomes between complex training group and control group, including **(A)** countermovement jump (CMJ) height, **(B)** squat jump (SJ) height. Note: the relative weight of each study in the analyses is indicated by the size of the green squares.

In addition, a moderate to large significant difference was noted between CT and control (between group, post analysis) for countermovement jump (SMD = 0.96, 95% CI = 1.64–0.29, *p* = 0.005, Figure 3A) and squat jump height performance (SMD = 1.58, 95% CI = 2.38–0.78, *p* < 0.001, Figure 3B). The relative weight of each study in the analyses varied between 10.4 and 23.1%, with moderate heterogeneity (*I*^2^ = 60.0–73.0%).

### Meta-Analysis Results for Change of Direction Performance

A moderate to large significant improvement was noted after CT (within group, pre-post analysis) for COD with ≤ 3 turns (SMD = 1.11, 95% CI = 0.33–1.88, *p* = 0.005) and COD with >3 turns (SMD = 1.24, 95% CI = 0.83–1.64, *p* < 0.001). The relative weight of each study in the analyses varied between 8.7 and 19.5%, with moderate to high heterogeneity (*I*^2^ = 26.0–77.0%).

**Figure 4:**
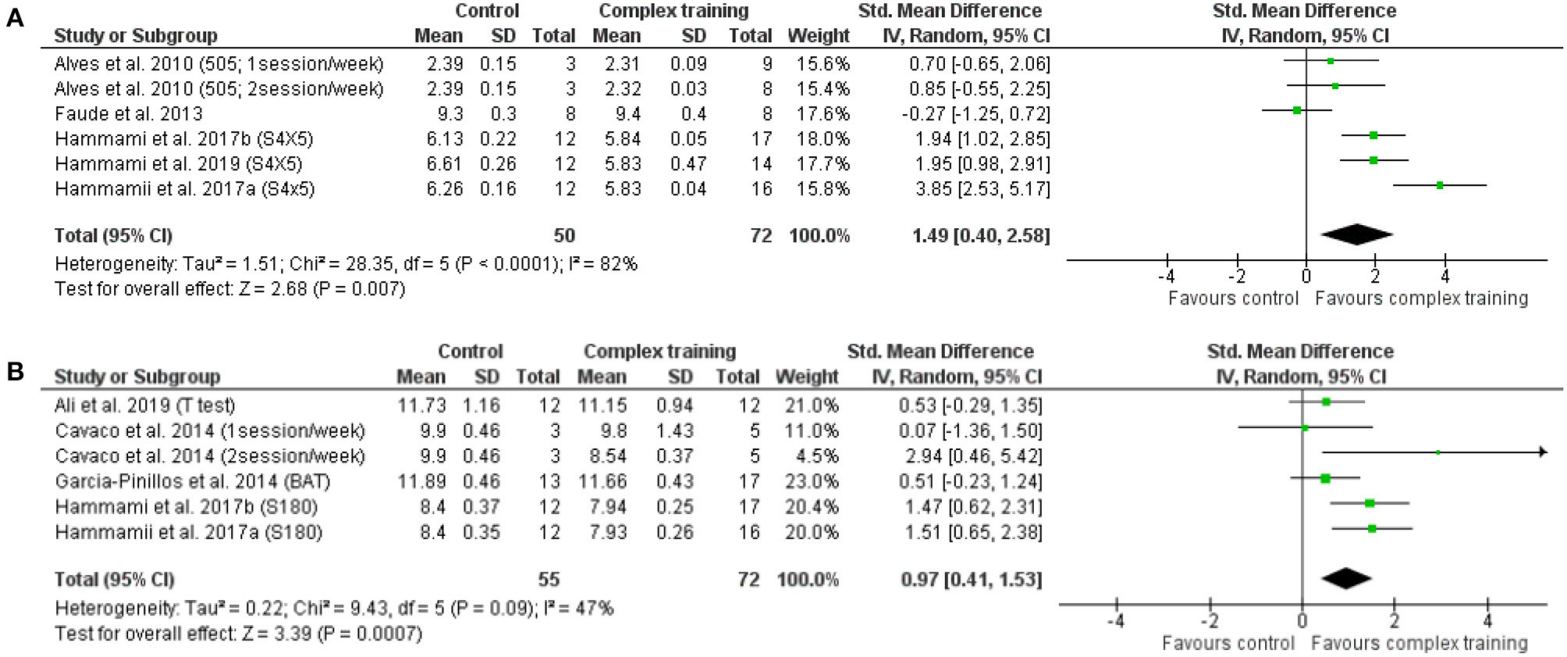
Forest plot of standardized mean difference in post-intervention outcomes between complex training group and control group, including **(A)** change of direction time with ≤ 3 turns, and **(B)** change of direction time with >3 turns. Note: the relative weight of each study in the analyses is indicated by the size of the green squares.

In addition, a moderate to large significant difference was noted between CT and control (between group, post analysis) for COD with ≤ 3 turns (SMD = 1.49, 95% CI = 0.40–2.58, *p* = 0.007, Figure 4A) and COD with >3 turns (SMD = 0.97, 95% CI = 0.41–1.53, *p* < 0.001, Figure 4B). The relative weight of each study in the analysis varied between 4.5 and 23.0%, with moderate to high heterogeneity (*I*^2^ = 47.0–82.0%).

### Subgroup Analyses

A subgroup analysis was performed based on age (< 18 vs. ≥18 years), playing level (professional vs. amateur), and duration of the training intervention (<8 vs. ≥8 weeks) as covariates (Table 5).

**Table 5:** Subgroup analyses of potential moderator factors for linear sprint time, vertical jump height and sprint with change of direction time.

	**Groups (*n*)**	**SMD (95% CI)**	***I*^**2**^**	***p***	***p*_**diff**_**
**5 m sprint**					
**Age**					
≥18	2	2.84 (−1.85–7.54)	95	0.24	0.85
<18	6	2.37 (1.21–3.53)	87	<0.001	
**Duration**					
≥8 weeks	6	2.69 (1.22–4.15)	92	<0.001	0.31
<8 weeks	2	1.82 (0.98–2.65)	0	<0.001	
**10 m sprint**					
**Age**					
≥18	2	0.21 (−0.45–0.87)	0	0.53	0.12
<18	4	0.81 (0.44–1.18)	0	<0.001	
**Playing level**					
Professional	5	0.75 (0.41–1.09)	0	<0.001	0.16
Amateur	1	0.00 (−0.98–0.98)	–	1.00	
**Duration**					
≥8 weeks	4	0.72 (0.34–1.09)	0	<0.001	0.63
<8 weeks	2	0.48 (−0.39–1.36)	46	0.28	
**20 m sprint**					
**Age**					
≥18	3	1.06 (−0.15–2.28)	80	0.09	0.32
<18	3	1.76 (1.11–2.42)	47	<0.001	
**Playing level**					
Professional	4	1.38 (0.49–2.27)	78	0.002	0.86
Amateur	2	1.55 (−0.03–3.13)	81	0.05	
**Duration**					
≥8 weeks	5	1.56 (0.76–2.36)	76	<0.001	0.19
<8 weeks	1	0.78 (−0.05–1.62)	–	0.07	
**30 m sprint**					
**Age**					
≥18	2	−0.13 (−0.79–0.52)	0	0.69	** <0.001**
<18	4	2.01 (0.97–3.06)	81	<0.001	
**Playing level**					
Professional	5	1.58 (0.46–2.71)	87	0.006	**0.03**
Amateur	1	−0.08 (−1.06–0.90)	–	0.88	
**Duration**					
≥8 weeks	4	1.64 (0.19–3.10)	90	0.03	0.34
<8 weeks	2	0.66 (−0.76–2.08)	78	0.36	
**CMJ**					
**Age**					
≥18	3	−0.71 (−1.43 to −0.35)	0	<0.001	0.57
<18	6	−0.99 (−1.79 to −0.19)	80	0.02	
**Playing level**					
Professional	7	−1.00 (−1.68 to −0.32)	76	<0.001	0.35
Amateur	2	−0.55 (−1.18–0.09)	0	0.09	
**Duration**					
≥8 weeks	4	−1.54 (−2.50 to −0.59)	79	0.002	**0.03**
<8 weeks	5	−0.40 (−0.80–0.01)	0	0.05	
**SJ**					
**Age**					
≥18	1	−0.82 (−1.74–0.10)	–	0.08	0.36
<18	5	−1.44 (−2.39 to −0.49)	80	0.003	
**Duration**					
≥8 weeks	3	−2.01 (−3.24 to −0.78)	79	0.001	**0.04**
<8 weeks	3	−0.64 (−1.17 to −0.11)	0	0.02	
**COD** **≤3 turns**					
**Age**					
≥18	1	0.66 (−0.35–1.68)	–	0.20	0.45
<18	5	1.19 (0.28–2.10)	80	0.01	
**Playing level**					
Professional	5	1.19 (0.28–2.10)	80	0.01	0.45
Amateur	1	0.66 (−0.35–1.68)	–	0.20	
**Duration**					
≥8 weeks	3	1.84 (1.02–2.66)	63	<0.001	**0.002**
<8 weeks	3	0.31 (−0.25–0.87)	0	0.28	
**COD** **>3 turns**					
**Age**					
≥18	2	0.82 (0.16–1.48)	10	0.01	0.13
<18	5	1.43 (1.00–1.86)	8	<0.001	
**Playing level**					
Professional	4	1.53 (1.12–1.95)	0	<0.001	**0.02**
Amateur	3	0.63 (0.01–1.24)	0	0.05	
**Duration**					
≥8 weeks	4	1.53 (1.12–1.95)	0	<0.001	**0.02**
<8 weeks	3	0.63 (0.01–1.24)	0	0.05	

*CI, confidence interval; CMJ, countermovement jump; COD, change of direction; I^2^, heterogeneity; p_diff_, test for subgroup difference; p, test for overall effect; SJ, squat jump; SMD, standardized mean difference. The bold values represents statistical significance between subgroups*.

Regarding the chronological age of players, a significant difference (*p* < 0.001, Table 5) in 30-m linear sprint time was noted between players ≥18 years (SMD = −0.13) and <18 years (SMD = 2.01), in favor of younger players, after CT.

For training programme duration, a greater improvement in countermovement jump (SMD = 1.54 vs. 0.40, *p* = 0.02), squat jump (SMD = 2.01 vs. 0.64, *p* = 0.04), COD with ≤ 3 turns (SMD = 1.84 vs. 0.31, *p* = 0.002), and COD >3 turns (SMD = 1.53 vs. 0.63, *p* = 0.02) was noted after ≥8 weeks compared to <8 weeks of CT (Table 5).

Regarding the competitive level of soccer players, a greater improvement in 30-m linear sprint time (SMD = 1.58 vs. 0.08, *p* = 0.03) and COD >3 turns (SMD = 1.53 vs. 0.63, *p* = 0.02) was noted in professional compared to amateur players after CT (Table 5).

### Adverse Effect of Complex Training Intervention

Among the included studies, no injuries related to CT were reported. One study (Spineti et al., 2019) reported the total RPE-load for CT and RT intervention. A total RPE-load of 25,201 was reported after 48 sessions of CT, while a total RPE-load of 25,364 was reported after RT.

## Discussion

Our meta-analyses indicated that compared to soccer training alone, CT induced moderate to large improvements in linear sprinting time, vertical jump height and sprinting with COD time in soccer players. The potentially beneficial effects derived from CT seem particularly applicable to younger players (<18 vs. ≥18 years, for linear sprinting), after longer training interventions (<8 vs. ≥8 weeks, for jumping and COD) and among players with greater competitive level (professional vs. amateur, for linear sprinting and with COD). Further, among the included studies, no injuries related to CT were reported. Overall, CT seems safe and effective compared to soccer training alone for the improvement of the physical fitness of soccer players.

The improvements in linear sprinting, vertical jumping and sprinting with COD performance following CT may be related to adaptative mechanisms similar to those induced by RT and PT alone, including maximal strength, hormonal milieu, structural, and neuromechanical adaptations, which may be potentiated through a cumulative post-activation performance enhancement effect induced with CT (Sale, 2002; Robbins, 2005; Carter and Greenwood, 2014). Indeed, several CT interventions included in this systematic review observed significant 1RM improvements in soccer players to a similar extent as RT (Faude et al., 2013; Brito et al., 2014; Spineti et al., 2016; Hammami et al., 2017a, 2019; Chatzinikolaou et al., 2018). As maximal strength is strongly related to sprint, jump, and COD in male soccer players (Wisløff et al., 2004), improvements in maximal strength may be an additional and relevant benefit derived from CT. In addition, improvements in sprint, jump, and COD performance after CT may be related to hormonal (Beaven et al., 2011; Ali et al., 2019) (e.g., testosterone increase) and cellular adaptations favorable to strength-power generation. For example, type IIx muscle fibers (i.e., those with greater contraction velocity, power, and rate of force development compared to type IIA and type I fibers) (Bottinelli et al., 1996; Harridge et al., 1996) may be favorably affected (i.e., greater preservation) by CT, even when compared to RT (Stasinaki et al., 2015). Indeed, an early study (Adams et al., 1993) reported that 19 weeks of heavy RT reduced percentage of type IIx fibers, whereas a more recent study (Stasinaki et al., 2015) reported a preservation of type IIx fibers after CT, similar to other studies that have included PT or CT (Macaluso et al., 2012, 2014; Grgic et al., 2020). Although not analyzed in the current study, the combination of RT and PT during CT may allow the summation of cellular and structural adaptations induced by RT and PT. Indeed, increased leg muscle volume was observed in soccer players after CT (Hammami et al., 2017a,b; Hammami et al., 2019). Although increases in muscle volume are usually achieved after RT interventions (ACSM, 2009; Krzysztofik et al., 2019), recent research suggest that PT may also induce significant hypertrophic effects (Grgic et al., 2020; Gumpenberger et al., 2020). Further, such structural adaptations after RT and PT seem not to interfere with neuromechanical adaptations (ACSM, 2009; Markovic and Mikulic, 2010). In fact, CT may favor energy transfer between concentric and eccentric muscle actions, providing better coordination and synchronization of active muscle groups to improve and enhance motor skills (Cronin et al., 2001; Robbins, 2005). Such improvements may include sprinting, vertical jumping and sprinting with COD performance, in addition to other motor skills enhancements such as kicking ability (Cavaco et al., 2014; García-Pinillos et al., 2014). Altogether, CT seems an effective training strategy for the enhancement of sprinting, jumping and sprint with COD. However, future studies should elucidate the effectiveness of CT on such outcomes among soccer players, particularly when compared to other training methods, including RT.

A cumulative post-activation performance enhancement effect induced with CT (Sale, 2002; Robbins, 2005) may help explain the performance enhancement (Tillin and Bishop, 2009; Petisco et al., 2019). Further, as CT combine higher (i.e., RT) and lower loads (i.e., PT) this may have optimized the force-velocity curve of soccer players (Cormie et al., 2011) by ensuring that the prescribed training addressed the two broad components of the continuum. Considering the relevance of the force-velocity spectrum parameters among soccer players (Jimenez-Reyes et al., 2020), it seems plausible that an optimization of the force-velocity spectrum may help to explain the improvements in performance after CT (Jiménez-Reyes et al., 2017; Jimenez-Reyes et al., 2019). Such a combination of high-load low-velocity and low-load high-velocity exercises during CT may favor recruitment of fast-twitch muscle fibers (Gołaś et al., 2016), which are particularly important during maximal-intensity and short-duration actions (e.g., vertical jumping) (Fry et al., 2003; Macaluso et al., 2012). In contrast to our findings, a previous meta-analysis (Bauer et al., 2019) did not observe jumping improvements after CT. However, Bauer et al. (2019) included studies involving a myriad of participants (e.g., schoolchildren, recreational trained students, untrained women, athletes from different sports) and compared the effects of CT with mixed training methods (e.g., RT, PT, mixed methods). In this sense, the results from our meta-analysis may offer a finding specific for soccer players (i.e., male soccer players), a finding that could have been potentially distorted amongst a wider population of athletes or the general population. On the other hand, extrapolation of current findings to other populations should proceed with caution.

Subgroup analyses were conducted to determine the effects of CT on physical fitness according to soccer player's chronological age, competitive level and duration of training programmes. Regarding the subgroup analysis according to the soccer players chronological age (<18 vs. ≥18 years), greater performance enhancements were noted after CT among players <18 years of age. The greater improvement among younger players may be due to the fact that players ≥18 years were already in the later stage of physical development and, hence, possessed a lower ceiling for further adaptation. In addition, players >18 years of age were likely to have performed RT and PT for a longer period of time (i.e., greater training age), experiencing diminishing return from CT (Carter and Greenwood, 2014) in the process. However, our subgroup analysis showed that professional players achieved greater performance improvements after CT compared to amateur players. Professional athletes have been shown to be better responders to post-activation performance enhancement (Carter and Greenwood, 2014). Indeed, professional athletes also tend to possess greater strength levels than amateur athletes, which might have enabled them to maximize the benefits of post-activation performance enhancement with CT (Seitz et al., 2014; Seitz and Haff, 2016; Suchomel et al., 2016). In addition, professional players' may possess a greater proportion of fast-twitch muscle fibers compared to amateur soccer players (Ostojic, 2004), rendering greater benefits from post-activation performance enhancement with CT (Seitz et al., 2014; Seitz and Haff, 2016; Suchomel et al., 2016). Furthermore, professional players may train with greater intensity, with professional athletes possessing greater intent to lift ballistically compared to non-professional athletes (Suchomel et al., 2018). In addition, CT was performed concurrently to soccer practices. It is possible that the number of soccer training sessions and games were greater in professional compared to non-professional players, with the former performing also greater number of sprints, jumps, and high-intensity actions. This may have also concurred to greater overall better neuromuscular adaptations in the professional vs. amateur athletes. Finally, regarding the subgroup analysis according to CT programme duration (<8 vs. ≥8 weeks), greater physical fitness improvements were noted after longer training interventions, in line with previous literature (Aagaard et al., 2001; Blazevich et al., 2007; Bolger et al., 2015). Although not surprising, current findings confirm that longer CT interventions may also induce larger gains in the physical fitness of male soccer players, particularly for jumping and sprints with COD.

Some limitations are acknowledged. Firstly, the findings of our systematic review suggest the absence of CT studies involving female soccer players. Therefore, current findings should not be simply extrapolated to female athletes. Future CT research should involve female soccer players to overcome this shortcoming in literature. Another major shortcoming noted in this systematic review is the lack of studies comparing CT to RT. Future research should focus on this comparison.

## Conclusion

The findings of this meta-analysis suggests that supplementing the regular soccer training sessions with CT improves athlete's physical fitness (i.e., sprinting, jumping, and COD ability) compared to soccer training alone. A longer duration of CT (≥8 weeks) seems to be optimal in improving the physical abilities of soccer players. Professional players and <18 years players may benefit more from CT program.

## Data Availability Statement

The original contributions presented in the study are included in the article/supplementary material, further inquiries can be directed to the corresponding author.

## Author Contributions

All authors made significant contributions, including preparation of the first draft of the manuscript, data collection, analysis of data, interpretation of data, and/or provided meaningful revision and feedback, read, and approved the final manuscript.

## Conflict of Interest

The authors declare that the research was conducted in the absence of any commercial or financial relationships that could be construed as a potential conflict of interest.
